# Transcriptome-wide analysis of the SCNT bovine abnormal placenta during mid- to late gestation

**DOI:** 10.1038/s41598-019-56566-w

**Published:** 2019-12-27

**Authors:** Guangqi Gao, Shenyuan Wang, Jiaqi Zhang, Guanghua Su, Zhong Zheng, Chunling Bai, Lei Yang, Zhuying Wei, Xiuying Wang, Xiao Liu, Ziru Guo, Guangpeng Li, Xiaohu Su, Li Zhang

**Affiliations:** 1 The State key Laboratory of Reproductive Regulation and Breeding of Grassland Livestock, Inner Mongolia University, Hohhot, 010070 China; 2 College of Life Science, Inner Mongolia University, Hohhot, 010070 China; 3 College of Life Science, Inner Mongolia Agricultural University, Hohhot, 010018 China; 4 State Key Laboratory of Stem Cell and Reproductive Biology, Institute of Zoology, Chinese Academy of Sciences, Beijing, 100101 China; 5 Inner Mongolia Radio and TV University, Hohhot, 010010 China; 6 Key Laboratory of Gene Engineering of the Ministry of Education, Guangzhou Key Laboratory of Healthy Aging Research and State Key Laboratory of Biocontrol, SYSU-BCM JointResearch Center, School of Life Sciences, Sun Yat-sen University, Guangzhou, 510275 China

**Keywords:** Developmental biology, RNA sequencing

## Abstract

The dysfunction of placenta is common in somatic cell nuclear transfer (SCNT) cloned cattle and would cause aberrant fetal development and even abortion, which occurred with highest rate at the mid- to late gestation. However, the mechanism of abnormal placentas was unclear. To analyze the transcriptome-wide characteristics of abnormal placentas in SCNT cloned cattle, the mRNA, lncRNA and miRNA of placental cotyledon tissue at day 180 after gestation were sequenced. A total of 19,055 mRNAs, 30,141 lncRNAs and 684 miRNAs were identified. Compared with control group, 362 mRNAs, 1,272 lncRNAs and nine miRNAs (six known and three novel miRNAs) were differentially expressed (fold change ≥ 2 and *P*-value < 0.05). The differentially expressed genes were functionally enriched in urea and ions transmembrane transport, which indicated that the maternal-fetal interactions were disturbed in impaired placentas. Furthermore, the competing endogenous RNAs (ceRNAs) networks were identified to illustrate their roles in abnormal placental morphology. The present research would be helpful to discover the mechanism of late gestational abnormality of SCNT cattle by provides important genomic information and insights.

## Introduction

The establishment of somatic cell nuclear transfer (SCNT) technology supplies a powerful impetus for the development of animal cloning. The wide-spread application of SCNT made the propagation of genetics and production of gene modified livestock time-saving and efficient^1^. Although the improvements of this technology have been made over the past two decade, the clone efficiency of SCNT cattle remains relatively low. Series research showed that the birth rate of cloned embryos was 5 to 15%^1–3^. At the early stage of embryonic implantation (30 to 90 days after gestation), the pregnant losses of SCNT embryo were associated with various placental abnormalities, such as hypoplasia of trophoblastic epithelium, alteration of basement membrane, degeneration of allantoic vessel and developmental retardation of the villous^3–6^. During the mid- to late gestation period, large offspring syndrome (LOS) was caused by hydrallantois and placenta megaly, even the lethality of fetus and its recipient^3,7–9^.

The correlation between placental gene expression variation and aberrant pregnancy were followed with interest for the developmental abnormality partly induced by placental disorder in SCNT cattle^10^. Placental array analysis showed that placental failure of cloned bovine to be associated with abnormal embryo-maternal communication during the peri-implantation period^11^. Salilew-Wondim *et al*. found that compared with artificial inseminated (AI) pregnancy, the alterations of gene expression in SCNT bovine placenta were more significant than *in vitro* embryo production (IVP) pregnancy at day 50 of gestation. Further analysis showed that the dysregulation of 9% of these genes was caused by transcriptional reprogramming error^12^. RNA-seq of cloned bovine extraembryonic tissue showed that differentially expressed genes between SCNT and AI leaded to the inhibition of trophoblast and placental development at both preimplantation and postimplantation stages^13^. Besides, the placental tissue microRNAs (miRNAs) analysis of deceased cloned calves suggested that the abnormal miRNA expression play a role at aberrant epigenetic reprogramming and late-fetal and/or neonatal lethality^14^.

Our previous work found that aberrant large abdominal circumference of recipient cows carrying SCNT fetuses at 150 to 200 days of gestation had a tight connection with hydrops allantois, placental hypertrophy and fetal overgrowth. In this study, abnormal placentas at mid- to late gestation were sampled and the mRNAs, lncRNAs and miRNAs expression profiles were detected through RNA-seq. Subsequently, the function of key genes, lncRNAs, miRNAs and their potential interactions on placental abnormalities were analyzed using bioinformatics. This study will expand our knowledge on aberrant SCNT bovine pregnancy and would be helpful to discover the mechanism of late gestational abnormality of SCNT cattle.

## Results

### The development of SCNT bovine fetus was affected by placental abnormality

**Figure 1:**
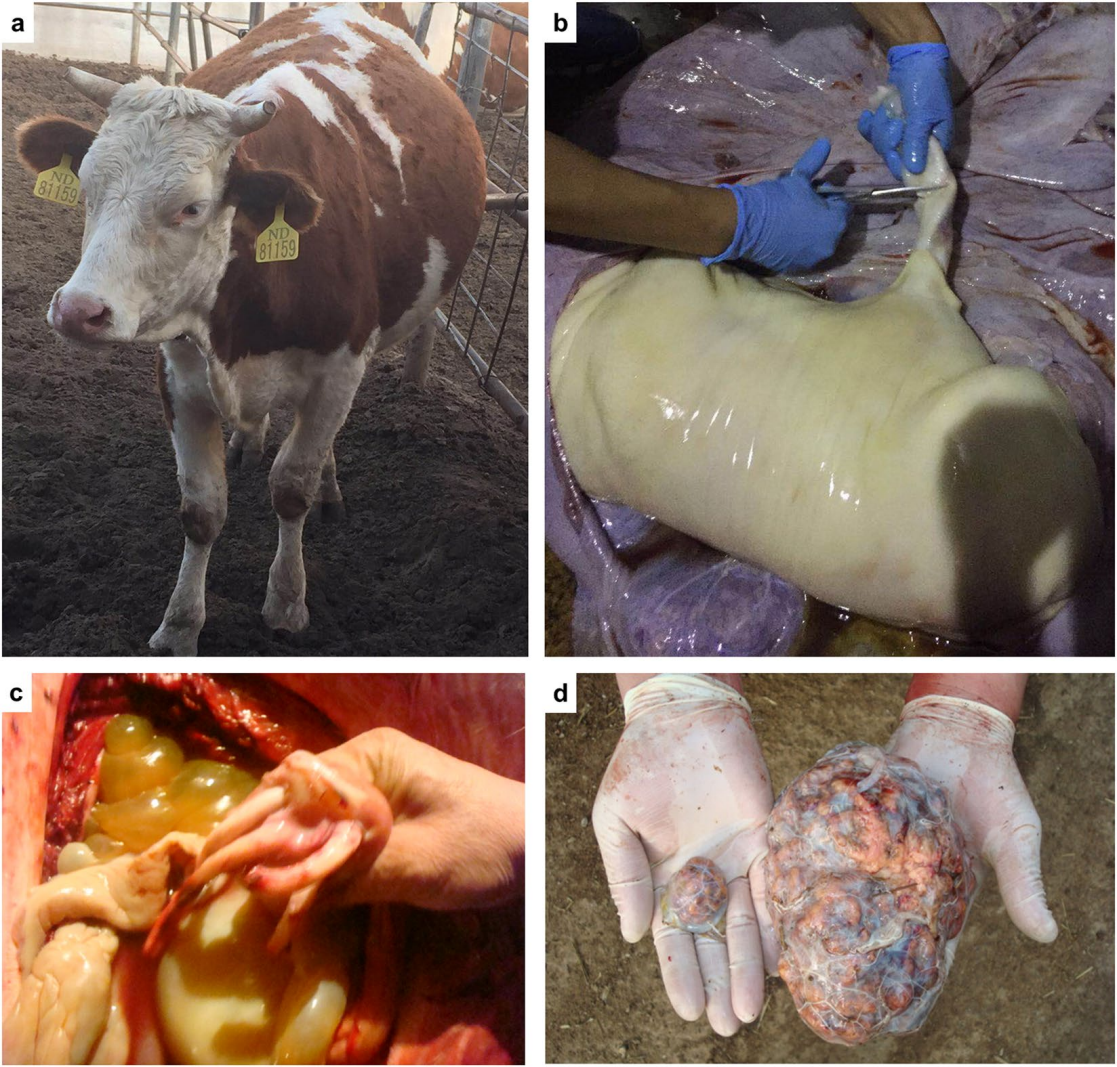
Clinical features of SCNT cattle during mid and late-pregnancy. (**a**) A case of SCNT fetus carried recipient cow with abnormally large abdominal circumference at 180 days of gestation. (**b**) A case of oversized SCNT bovine fetus. **(c)** The umbilical cord enlargement of the SCNT bovine fetus. (**d**) Placental hypertrophy and abnormal size of placental cotyledons.

Our previous studies showed that the bovine embryos derived from artificial technology (especially asexual reproduction) with high abortion rate throughout the pregnancy process, particularly during the mid and late-gestation. According to our research data from 2008 to 2018, the rate of abortion was highest at 150 to 200 days of gestation. In one of experiments, the abortion rate reached to 24.00% in this period (unpublished data). The major symptom was that the abdominal circumferences of the recipient cows were aberrant large and aborted subsequently (Fig. 1a). Most of fetuses were oversized (Fig. 1b). A more extreme example was that the recipient cow delayed delivery for 30 days and signs of production appeared until 315 days after gestation. A huge calf was produced via caesarean section with the birth weight of 78 kg, which was 3 times to the normal calves (unpublished data). Unfortunately, this calf died less than a hundred days after birth.

The SCNT bovine abortions caused by LOS were also accompanied by placental abnormalities, which usually represented as enlargement of the umbilical cord, placental hypertrophy and abnormal size of placental cotyledons (Fig. 1c,d). In order to investigate the abnormal placentas at RNA level, we took out the fetuses and placentas after slaughter the recipients when the recipient cow was experientially observed to show abnormal abdominal circumference at day 180 ± 2 of gestation.

A total of 40 cows were used as recipients and 32 of which were estrous-synchronized. These recipients were transferred with SCNT (23 recipient cows) or *in vivo* produced embryos (9 recipient cows). At day 45 of gestation, the pregnancy rates of SCNT and *in vivo* produced embryos were 39.13% and 55.56%, respectively. At day 180 of gestation, the pregnancy rates of SCNT and *in vivo* produced fetuses were 13.04% and 55.56%, respectively (Table 1).

**Table 1:** Statistics on pregnancy at gestation days after embryo transplant.

Gestation day	SCNT cloned		*In vivo* derived	
	Pregnancy number	Pregnancy rate	Pregnancy number	Pregnancy rate
Day 7	23	—	9	—
Day 45	9	39.13%	5	55.56%
Day 180	3	13.04%	5	55.56%

The days of gestation were counted starting at the day of the heat (Day 0). Embryos are transferred at Day 7. The line of Day 7 indicates recipient cows numbers used for embryo transplant.

### Sequencing and mapping of mRNAs and lncRNAs

In order to develop a comprehensive catalogue of mRNAs and lncRNAs of bovine placentas, trascriptome data sets were generated by RNA-seq. Illumina sequencing of bovine placental cotyledons tissues yielded a total of 708,477,746 raw reads. The Phred Quality Score of the samples were more than 91% for Q30. Clean data included 133,443,802 reads at average and the minimum Q30 of clean data was 92.03%. Subsequently, the clean reads were mapped into the bovine reference genome using HiSAT2^15^. The average mapping rate of the five samples was 94.11% and the unmapped rates were between 5.51% and 6.82%. Detail summary of the sequencing results were showed in Table S1, which could indicate the high quality of transcriptome sequencing data with suitable mapping. A total of 19,055 mRNAs were recognized from these data. Furthermore, after a series of basic and coding potential screening, 30,141 lncRNA loci were identified by overlapped the predicted results from CNCI, CPC, PFAM and CPAT.

### Identification of miRNAs

To identify miRNAs of bovine normal and abnormal placentas, five small RNA libraries were constructed and sequenced independently. A total of 138,867,057 raw reads were generated. And 113,588,641 clean reads were obtained, which account for more than 77% of the raw data. Among five individual libraries, the minimum rates of removed reads were at a suitable level and the Q30 of raw or clean data were high enough to indicate the high-quality of small RNA sequencing (Table S2). The length of most sequences was distributed at the range of 21–23 nt and the highest percentage was 22nt, which is consistent with the common size of miRNAs. The total clean reads between 15–35 nt were aligned with the bovine genome using Bowtie software. The average perfect match rate was 72.70%. In total, 488 known and 196 novel miRNAs were identified using miRBase (Release 21) and miRDeep2^16^.

### Identification of differentially expressed mRNAs, lncRNAs and miRNAs between bovine normal and abnormal placentas

**Figure 2:**
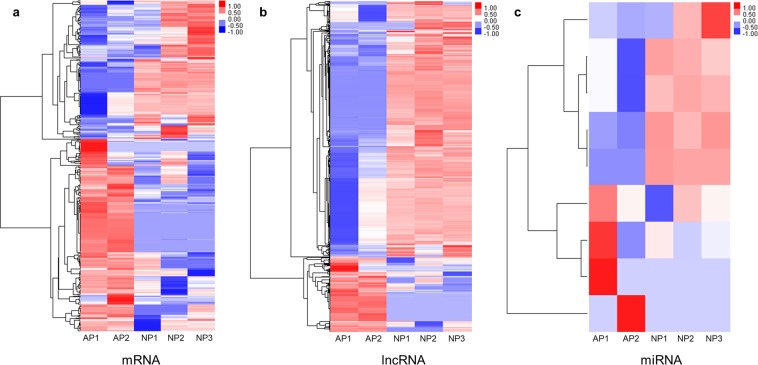
Hierarchical clustering of mRNA, lncRNA and miRNA expression patterns compared between abnormal (AP) and normal placentas (NP). Expression values are represented in shades of red and blue, indicating expression above and below the mean expression value, respectively.

The identification of differentially expressed mRNAs, lncRNAs and miRNAs were calculated using edgeR with the threshold of fold change ≥2 and P-value < 0.05. 362 mRNAs, 1,272 lncRNAs and 9 miRNAs (6 known and 3 novel miRNAs) were found to be differentially expressed between abnormal and normal groups. Compared with normal group, 208 mRNAs, 283 lncRNAs and 4 miRNAs were up regulated, as well as 154 mRNAs, 989 lncRNAs and 5 miRNAs were down regulated in abnormal group. Expression patterns of mRNA, lncRNA and miRNA were shown by hierarchical clustering (Fig. 2). Then these differentially expressed non-coding RNAs (ncRNAs) were intersected with predicted target genes. As a result, 2,836 and 1,241 genes targeted with lncRNA and miRNA were screened out, respectively (Tables S3 and S4).

### Functional enrichment analyses of differentially expressed ncRNAs target genes and differentially expressed mRNAs

To investigate the function of differentially expressed lncRNAs and miRNAs, the enrichment analyses of their target genes were performed. For target genes of differentially expressed lncRNAs, substance metabolism pathways were significantly enriched, including metabolic pathways (KO01100), carbon metabolism (KO01200) and glycerol phospholipid metabolism (KO00564). Besides, transcription associated functions were also high representation, such as Poly(A) RNA binding (GO: 0044822), catalytic step 2 spliceosome (GO: 0071013), mRNA splicing via spliceosome (GO: 0000398), RNA splicing (GO: 0008380), mRNA processing (GO: 0006397) and RNA binding (GO: 0003723). For target genes of differentially expressed miRNAs, the most significantly enriched pathway was ECM-receptor interaction (KO04512), which involved functions of tissue structural maintaining and organ morphogenesis. Moreover, hormone related pathways could also be found, such as ovarian steroidogenesis (KO04913), GnRH signaling pathway (KO04912), estrogen signaling pathway (KO04915) and aldosterone synthesis & secretion (KO04925). The results suggested that the abnormal ncRNAs expression may affect the material metabolism and hormone secretion of bovine placenta via regulate target genes.

**Figure 3:**
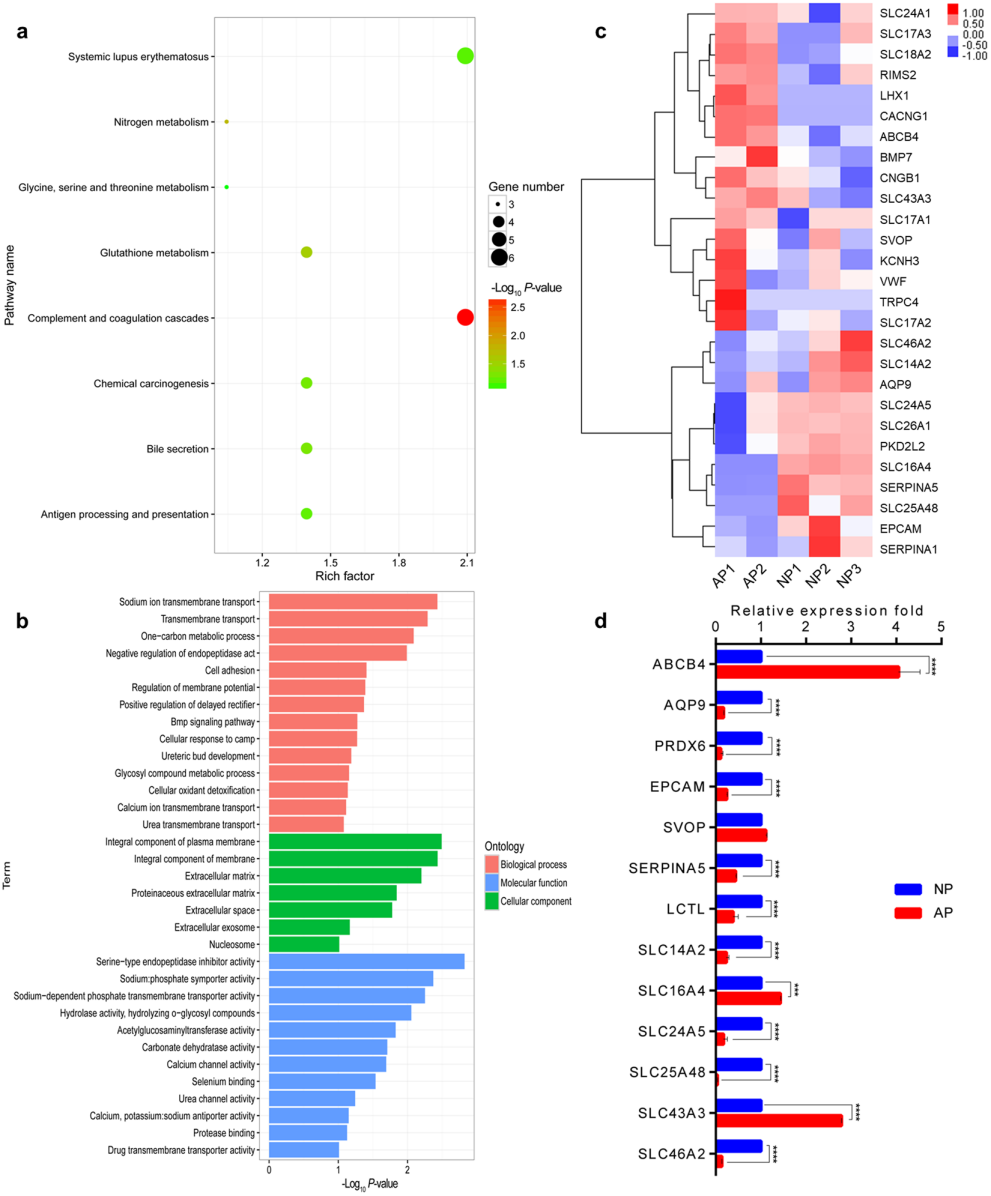
The functional enrichment of the differentially expressed genes between between abnormal (AP) and normal placentas (NP). (**a**) The KEGG pathway enrichment of the DEGs. (**b**) The GO enrichment of the DEGs. (**c**) Expression patterns of selected genes associated with urea and ions transmembrane transport. (**d**) Candidate genes validated by q-PCR. ***P < 0.001, ****P < 0.0001.

As the expression of mRNA is more directly related to the biological characters, the function of differentially expressed mRNAs was analyzed by KEGG and GO enrichment. A total of 8 KEGG pathways were enriched by the 362 differentially expressed genes (DEGs), of which the complement and coagulation cascades pathway was most significant (Fig. 3a). It was noteworthy that these DEGs were also functionally enriched in GO categories for the transmembrane transport of urea and ions (Fig. 3b). In the categories of ureteric bud development (GO: 0001657), urea transmembrane transport (GO: 0071918) and urea channel activity (GO: 0015265), the expressions of *BMP7* and *LHX1* were up-regulated in abnormal group, while *SLC14A2*, *EPCAM* and *AQP9* were down-regulated (Fig. 3c). In the categories associated with ion transmembrane transport, 22 genes were involved. The expressions of 14 genes (*SLC43A3*, *SVOP*, *VWF*, *RIMS2*, *ABCB4*, *SLC18A2*, *CNGB1*, *SLC24A1*, *KCNH3*, *SLC17A2*, *SLC17A1*, *SLC17A3*, *CACNG1*, *TRPC4*) were up-regulated in abnormal group, and the other 8 genes (*SLC16A4*, *PKD2L2*, *SLC25A48*, *SLC24A5*,*SLC26A1*, *SERPINA1*, *SLC46A2*, *SERPINA5*) were down-regulated (Fig. 3c). To confirm the gene expression patterns, half of these genes were randomly selected to be validated by q-PCR. The results were in concordance with the RNA-seq data (Fig. 3d).

### The interaction network between ncRNAs and their target mRNAs

**Figure 4:**
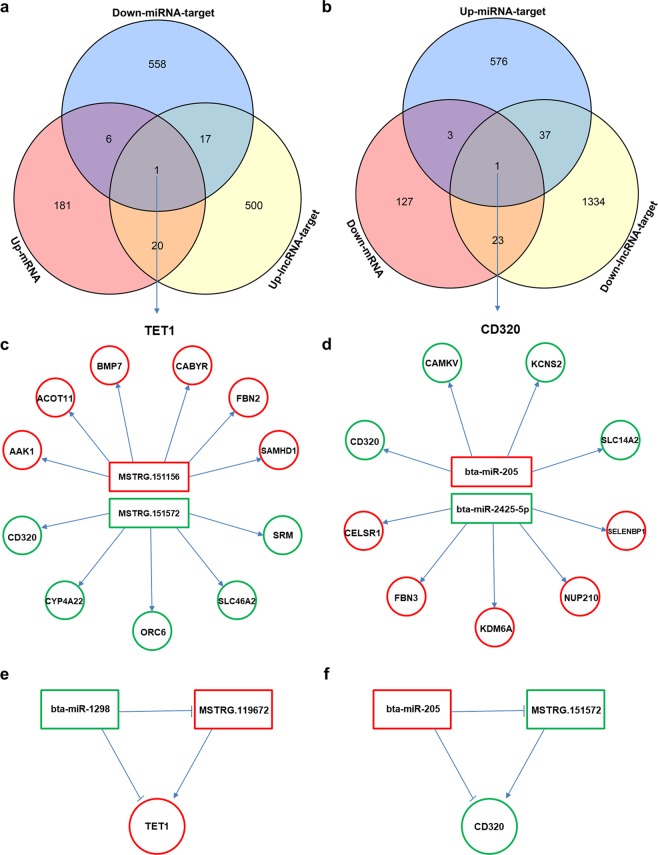
The interaction network between mRNAs and their target non-coding RNAs. (**a**) Overlap of up-regulated lncRNAs, up-regulated DEGs and down-regulated miRNAs in the abnormal placentas. (**b**) Overlap of down-regulated lncRNAs, down-regulated DEGsandup-regulated miRNAs in the abnormal placentas. (**c**) lncRNAs(in box) that potentially regulate most of the DEGs (in circle). Red indicates up- regulation, green indicates down- regulation. (**d**) miRNAs(in box) that potentially regulate most of the DEGs (in circle). Red indicates up- regulation, green indicates down- regulation. (**e**) The ceRNA interactions of bta-miR-1298-MSTRG.119672-*TET*1. (**f**) The ceRNA interactions of bta-miR-205-MSTRG.151572-*CD*320.

DEGs and their corresponding differentially expressed lncRNAs or miRNAs were selected according to the regulation relationship and expression trend between mRNAs and ncRNAs. In the abnormal group, target genes of up-regulated lncRNAs or down-regulated miRNAs were overlapped with up-regulated DEGs, as well as the target genes of down-regulated lncRNAs or up-regulated miRNAs were overlapped with down-regulated DEGs (Fig. 4a,b, Table 2). Based on the expression pattern of competing endogenous RNAs (ceRNAs), these overlapped DEGs were more likely to be regulated by lncRNAs or miRNAs^17^. Most of the up- and down-regulated DEGs were probably regulated by MSTRG.151156 and MSTRG.151572, respectively (Fig. 4c, Tables 2 and S5). Simultaneously, bta-miR-205 and bta-miR-2425-5p targeted more DEGs either (Fig. 4d, Table S5). It was worth noting that *TET1* (up-regulated in abnormal group) and *CD320* (down-regulated in abnormal placentas) were under the regulation of both lncRNA and miRNA (Fig. 4a,b), such as bta-miR-1298-MSTRG.119672-*TET*1 and bta-miR-205-MSTRG.151572-*CD*320 ceRNA networks (Fig. 4e,f). MSTRG.119672 serves as ceRNA to up regulate *TET*1. As a negative regulator, bta-miR-205 inhibits the expression of *CD*320.

**Table 2:** The differentially expressed candidate genes and their target non-coding RNAs in bovine placentas.

Gene		target lncRNA		target miRNA	
BMP7	↑	MSTRG.119672	↑	—	
		MSTRG.161616	↓	—	
		MSTRG.151156	↑	—	
		MSTRG.236813	↑	—	
SLC14A2	↓	—		bta-miR-205	↑
RIMS2	↑	MSTRG.196921	↓		
ABCB4	↑	MSTRG.161616	↓		
		MSTRG.195717	↓		
KCNH3	↑	MSTRG.181130	↓		
TRPC4	↑	—		Novel_97	↑
SLC16A4	↓	—		bta-miR-1298	↓
SLC46A2	↓	MSTRG.151572	↓	—	
SERPINA5	↓	MSTRG.181130	↓	—	

The arrows represent up or down expressions of RNAs in the abnormal placentas tissues.

## Discussion

SCNT is one of the most useful embryo engineering techniques in basic research and livestock breeding. However, the bottleneck of low developmental efficiency and high incidence of abnormalities of SCNT offspring limits its development. Abortion of SCNT-derived fetuses usually caused by placental hypertrophy, allantoichydrops and placental edema at day 150–200 of pregnancy, and the placental hypertrophy is almost the universal complication of SCNT fetal death^18^. In this study, we focused on the SCNT bovine oversized placentas to understand the mechanism of abnormally SCNT bovine pregnant during mid to late gestation through the transcriptome analysis.

### Clinical features of abnormal pregnant SCNT cattle during mid and late-gestation

During 2008 to 2018, a total of approximately 4,000 SCNT, *in vitro* fertilization (IVF) and *in vivo* produced bovine embryos were transferred by our team. We found that LOS and concomitant placental hypertrophy were occurred mainly in SCNT fetuses and some of IVF fetuses. About 1/4 recipient cows which carried SCNT fetuses exhibited symptom of larger abdominal circumference and eventually lead to the death of the recipients and the offspring. This problem has always been one of the great difficulties in animal husbandry^19^. Similar as Liu’s study, we found that this problem was donor cell-dependant^9^. In this study, several recipients exhibited the symptom with large abdominal circumference. And RNAs extracted from these abortive placentas failed to meet the criteria of transcriptome sequencing. Finally, we slaughtered the abnormal pregnant recipients to obtain hypertrophic placentas.

### Functional affection of abnormal expressed genes of SCNT bovine placenta

Understanding of the transcriptome-wide landscape is conducive to discover the mechanism associated with placental morphological and functional changes. Similar with previous transcriptome or proteome studies, we also found that DEGs between abnormal and normal placentas were functionally enriched in complement and coagulation cascades pathway^20,21^. The result suggested that the regulatory changes of this pathway might influence the normal development of placentas. The functional GO terms of urea and ions transporting across membrane were associated with maternal-fetal interaction, in which a series of DEGs were involved.

In the categories of ureteric bud development and urea transport, *BMP7* and *LHX1* were up-regulated in abnormal placentas. *BMP7* was shown to affect embryonic cloacal cavity separates, development of urethral system, as well as placental abnormalities^22,23^. Complete inactivation of *BMP7* were found to restore ureteric bud outgrowth and branching^24^. Similarly, *LHX1* was also considered to be associated with dysplasia of urogenital system development^25^. On the contrary, *AQP9*, *EPCAM* and *SLC14A2* were identified to be expressed significantly lower in abnormal placentas by both RNA-seq and q-PCR. *AQP9* encodes a water channel protein which participates in stimulating urea transport. Up-regulation of *AQP9* were required when organismic repairation of pathological placental changes^26^. The abnormal amniotic fluid was considered to be related with the expression alterations of *AQP*s to in human, in which AQP9 played a role in the urea uptake and elimination across the placenta^27^. EPCAM were generally defined as a potential tumor marker and immunotherapy target, it was also suggested that inactivation of this gene could lead to prominent placental abnormalities. Placentas of *EPCAM*^−/−^ mice exhibited thin labyrinthine layers and lacking of vascularity and induced early embryonic lethality^28^. However, *EPCAM*^+/−^ mice were normal. SLC14A2 is one of the two major subgroups of the SLC14A urea transporter family, which is mainly expressed in kidney^29^. Down-regulation of *SLC14A2* in abnormal placentas might cause difficulty of urea excreting. Therefore, abnormal expression of these genes in placentas might impact the transport of urea in SCNT bovine fetuses.

In the category of ions transmembrane transport, most of DEGs in this study were members of solute carrier family. Expression changes of these genes in abnormal placentas might be associated with maternal-fetal substances circulations for their crucial roles in transport of ions and drugs. The aberrant expression pattern of these genes not only causes the deliver obstruction of various substances including urate, but also may affect the placenta morphology and maternal-fetal interaction, which further aggravates placental dysfunction.

### Effecting of ceRNA for SCNT bovine placental abnormity

Abnormal gene expression in SCNT bovine placenta could be attributable to the epigenome features of the somatic cell genome caused by the inappropriate reprogramming in SCNT embryos^30,31^. Besides, it is known that ncRNAs play diverse roles in regulating gene expression. In ceRNA network, miRNAs are usually considered to play negative regulations on mRNA, while lncRNAs can act as decoys of miRNAs to modulate gene expression^32,33^. Therefore, the expression of target lncRNAs is more similar with their corresponding genes.

In this study, some DEGs were likely to be regulated by lncRNAs or miRNAs, for the target genes of up-regulated lncRNAs or down-regulated miRNAs were overlapped with up-regulated DEGs, and the target genes of down-regulated lncRNAs or up-regulated miRNAs were overlapped with down-regulated DEGs. Two gene sets were obtained by above strategy, in which *TET1* and *CD320* were under the regulation by both lncRNA and miRNA. *TET1* is a member of the TET family, the function contains the regulation of DNA methylation in mouse embryonic stem cells, placental trophoblasts and pathological placental tissue^34–37^. The expression of *TET1* was under the negative regulation of miRNAs in cancer cells, such as miR-29, miR-26a, miR-767, miR-494 and miR-520b^38^. In current study, *TET1* was found to be regulated by bta-miR-1298 and a lncRNA MSTRG.119672 in bovine placenta. In addition, the expression of *CD320* was potentially co-regulated by bta-miR-205 and MSTRG.151572. CD320, also known as TCblR, is a transcobalam in receptor which is expressed in placenta with high quantities and mediates cobalamin (vitamin B12) maternal-fetal transport^39^. Impaired placental vascularization and endothelial dysfunction are considered to be associated with the concentration of placenta-related parameters in the circulation. Therefore soluble form of CD320 in serum was identified to be a potential biomarker for evaluating pregnancy risk^40,41^. We surmise that placental abnormity of SCNT cattle might be associated with the aberrant maternal-fetal transport of vitamin B12 by the altered expression of *CD320* and/or its target ncRNAs.

## Conclusions

In this study, we present the transcriptome-wide data of abnormal placental cotyledon tissues from SCNT cloned cattle at day 180 of gestation. The comparative analyses of mRNA, lncRNA, miRNA and ceRNA provide important genomic information and insights for further discovering the mechanism of abnormally SCNT bovine pregnant during late gestation.

## Methods

### Ethics approval and consent to participate

All experimental procedures and sample collections were conducted in accordance with the guidelines of the Inner Mongolia University Animal Care and Use Committee. The bovine ovaries used in this study were collected with permission of the Hohhot slaughterhouse. Experimental protocols were approved by the Institutional Animal Care and Use Committee at Inner Mongolia University.

### Design of experiments

The donor cell line used in this study was designedly selected which could lead to higher rate of placental abnormalities according to our previous research. The tissues derived from *in vivo* produced embryo were set as control group. The abnormal and normal placentas were sampled at day 180 ± 2 of pregnancy. These samples were used for RNA-seq and quantitative real-time PCR (q-PCR) analyses.

### Production of SCNT and *in vivo* embryos

In this study, fetal fibroblast of male Chinese Luxi cattle was used as donor for SCNT. The SCNT procedure was described as previously reported^15^. The protocols of nuclear transfer, fusion, activation, and embryo *in vitro* culture were followed as Wu *et al*.^16^. For *in vivo* embryo collection, donor cows (Chinese Simmental) were superovulated and inseminated with frozen/thawed semen from one Chinese Luxi bull with proven fertility. Morulae and blastocysts were collected by uterine flushing at day 7 or 8 after artificial insemination by routine nonsurgical procedure. Both SCNT and *in vivo* produced embryos were evaluated under a stereomicroscope and only morphologically intact embryos were selected for transfer.

### Embryo transfer and pregnancy detection

The treatment of estrus synchronization was carried out according to previous^17^. Briefly, Simmental heifers of 18–20 month-old were chosen as recipients. At day 0 of estrus synchronization, EAZI BREED CIDR (Pfizer Pty, New Zealand) was used. At day 9, the recipients were injected with 0.5 mg cloprostenol (prostaglandin F_2α_) (Ningbo second hormone factory, China). At day 11, CIDR was removed. The recipients were observed estrus conditions at day 12–13. Each of estrous-synchronized recipient cow was transferred one or two blastocysts non-surgically at day 7 after estrus. Pregnancy was first detected by trans-rectal ultrasound at around 45 days after estrus, and placental samples were collected at 180 days after estrus.

### Sample collection

At day 180 of gestation, three pregnant recipients which carried SCNT fetuses presented overgrowth of abdominal circumference compared to others. And the abnormal placental cotyledons were separated by caesarean section. In addition, three normal placental samples from *in vivo* produced embryo carrier recipients were collected as controls. These samples were snap-frozen in liquid nitrogen and stored for use towards the subsequent generation of RNA libraries.

### RNA sequencing

Prior to the transcriptome sequencing, total RNA of the collected bovine placental tissues was extracted using Trizol reagent (Invitrogen, USA) and the quality was determined using agarose electrophoresis, Spectrophotometer and Agilent 2100 RNA Nano 6000 Assay Kit(Agilent Technologies, USA). The extracted RNAs were stored at −80 °C before the next use.

For mRNA and lncRNA sequencing, the RNA libraries of each sample were constructed separately. After removing ribosomal RNA by Ribo-Zero^TM^ Gold Kits (Epicentre, USA), libraries were prepared by select different index Tags according the manufacturer’s instructions of NEB Next Ultra Directional RNA Library Prep Kit for Illumina (NEB, USA).Then these libraries were sequenced on an Illumina Hiseq X ten system in PE150 mode.

For miRNA sequencing, small RNA fragments of 15–35 nt were isolated from total RNA, then ligated with adaptors and synthesized to cDNA for amplification. Then the prepared libraries were sequenced on an Illumina Hiseq. 2500 system in SE50 mode.

### Quality control of sequencing data

The raw data were filtered under a series of steps as follow. For both RNA and miRNA data, low quality reads (>15% of bases whose Phred scores were ≤ 19) were removed. Then adapter-containing reads (the adapter sequence > 5 bp), unknown base calls (N) with the rate > 5% and rRNA matched reads were trimmed out from RNA raw data. For the raw data of miRNA, unknown base calls (N) with the rate > 10%, reads without 3’ adapters or insert fragments, reads containing poly-A/T or the length was not within the required range, were filtered to generate clean data. The Phred Quality Score (Q30) was calculated to assess the qualities of both raw and clean data. All subsequent analyses were based on the clean data.

### Transcriptome assembly

The reference bovine genome and the annotation file were downloaded from ENSEMBL database (http://www.ensembl.org/index.html). And Clean Data were mapped to the reference genome using HISAT2 (v2.0.5) (http://ccb.jhu.edu/software/ hisat2/index.shtml). The mapped reads of each sample were assembled by using StringTie (v1.3.2d) with the parameter of -G ref.gtf -rf–l^18,19^.

### Identification of lncRNA and miRNA

Bovine lncRNAs were identified from the assembled transcripts under the following conditions: (1) transcripts with length < 200 bp were removed; (2) transcripts with exon number < 2 were removed; (3) transcripts of all samples with reads coverage < 5 were removed; (4) compared with the annotation file of the species to screen the known mRNA and other non-coding RNA (rRNA, tRNA, snoRNA and snRNA) using the gffcompare software. (5) according to the information of class_code (“u”, “i”, “x”), the potential lincRNA, intronic lncRNAand anti-sense lncRNA were screened; (6) transcripts without coding potentials were removed byco-analysis of Coding-Non-Coding Index (CNCI), Coding Potential Calculator (CPC), PFAM database and Coding Potential Assessment Tool (CPAT)^20^.

The basic idea of known miRNA identification is to get overlap (100%) between genome location of mapping reads and the genome location of reference miRNA. If genome annotation data can be found in miRBase (Release 21), it just needs to get overlap by software Bed tools (v2.17.0)^21^. Otherwise, we will firstly map the reference miRNA to the reference genome to obtain location information, then get overlap. After excluded reads that mapped to known miRNA/ncRNA/repeat region/mRNA region, the remained reads were used to predict novel miRNA for animal by using software miRDeep2^22^. The key of identification was the hairpin structure formation of reads stack and possibility and stability evaluation.

### Quantification of RNAs expression level

For mRNAs and lncRNAs, read counts were counted by HTSeq^23^. FPKM (Fragments Per Kilobase Millon Mapped Reads) were calculated to represent the expression level in each sample^24^. For miRNAs, RPM (Reads Per Million total reads) values were considered as normalized count of sample, and can be directly used in inter-library comparison.

### Target gene prediction of lncRNA and miRNA

For lncRNAs, mRNAs were selected with High Spearman correlation coefficient (P ≥ 0.9) as the *trans*-targets. And the mRNAs with distance less than 50 kb were selected as the *cis*-targets. For miRNAs, miRanda (3.3a) were used to predict targets of known or novel miRNA. The principle of miRanda prediction was on the basis of seed region sequence alignment. Results would be filtered by parameters -sc 160 -en −20.

### Differential expressed RNAs and functional enrichment analyses

The differentially expressed RNAs (mRNAs, lncRNAs and miRNAs) between abnormal and control placental cotyledons were calculated by edgeR with P < 0.05 and |log_2_ ratio| ≥ 1. The enrichment analyses of KEGG (Kyoto Encyclopedia of Genes and Genomes) (https://www.kegg.jp/) and GO (Gene Ontology) (http://www.geneontology.org/) were performed by using DAVID (The Database for Annotation, Visualization and Integrated Discovery) (https://david.ncifcrf.gov/) under the background of *Bostaurus* species with the default parameters.

## Supplementary information

Supplementary information

## Data Availability

All data generated or analyzed during this study are available from the corresponding author upon reasonable request.
